# Effectiveness of Strict vs. Multiple Use Protected Areas in Reducing Tropical Forest Fires: A Global Analysis Using Matching Methods

**DOI:** 10.1371/journal.pone.0022722

**Published:** 2011-08-16

**Authors:** Andrew Nelson, Kenneth M. Chomitz

**Affiliations:** 1 International Rice Research Institute, Metro Manila, Philippines; 2 Independent Evaluation Group, World Bank, Washington, D. C., United States of America; University Copenhagen, Denmark

## Abstract

Protected areas (PAs) cover a quarter of the tropical forest estate. Yet there is debate over the effectiveness of PAs in reducing deforestation, especially when local people have rights to use the forest. A key analytic problem is the likely placement of PAs on marginal lands with low pressure for deforestation, biasing comparisons between protected and unprotected areas. Using matching techniques to control for this bias, this paper analyzes the global tropical forest biome using forest fires as a high resolution proxy for deforestation; disaggregates impacts by remoteness, a proxy for deforestation pressure; and compares strictly protected vs. multiple use PAs vs indigenous areas. Fire activity was overlaid on a 1 km map of tropical forest extent in 2000; land use change was inferred for any point experiencing one or more fires. Sampled points in pre-2000 PAs were matched with randomly selected never-protected points in the same country. Matching criteria included distance to road network, distance to major cities, elevation and slope, and rainfall. In Latin America and Asia, strict PAs substantially reduced fire incidence, but multi-use PAs were even more effective. In Latin America, where there is data on indigenous areas, these areas reduce forest fire incidence by 16 percentage points, over two and a half times as much as naïve (unmatched) comparison with unprotected areas would suggest. In Africa, more recently established strict PAs appear to be effective, but multi-use tropical forest protected areas yield few sample points, and their impacts are not robustly estimated. These results suggest that forest protection can contribute both to biodiversity conservation and CO2 mitigation goals, with particular relevance to the REDD agenda. Encouragingly, indigenous areas and multi-use protected areas can help to accomplish these goals, suggesting some compatibility between global environmental goals and support for local livelihoods.

## Introduction

Tropical deforestation accounts for between one fifth and one quarter of the total human contribution to greenhouse gases [1], [2], and 80% of emissions from the least developed countries. (Data for 2005, including land-used change and forestry, from CAIT 8.0.) Reduction of deforestation therefore contributes to climate change mitigation and may also provide development benefits [3], [4], [5]. The REDD (Reduced Emissions from Deforestation and Degradation) agenda seeks to integrate deforestation reduction into the global climate regime under the United Nations Framework Convention on Climate Change, rewarding countries that reduce forest emissions [3], [6].

Although the REDD agenda is new, the forest protection agenda is not. Conservation and sustainable management of forests have been motivated by biodiversity and livelihood concerns for decades. Where deforestation is a threat to biodiversity, successful conservation or sustainable management efforts will have a side benefit of reducing forest carbon emissions. This is especially salient in the humid tropical forests, where deforestation rates and carbon densities are both high. So an evaluation of the effectiveness of past conservation efforts can inform the design of interventions to promote REDD.

Among conservation interventions in tropical forests, the establishment of protected areas has been the most prominent and best funded [5]. The Global Environment Facility says that its investments in protected areas include $1.6 billion of its own resources and $4.2 billion in cofinancing; much of this has been implemented through the World Bank. Protected areas have expanded rapidly in recent years [7] and now cover around 27.1 percent of the tropical forest estate. (Authors' calculation. Boundary and area data are not available for a small percentage of protected areas, so this may be a conservative estimate.) In many ways they provide a model for broader classes of intervention, since most efforts to reduce deforestation will involve some kinds of restrictions on land use practices [5].

Yet there is considerable uncertainty and controversy over the impacts and effectiveness of protected areas. Views on their environmental effectiveness have see-sawed. In the 1990s, protected areas were often characterized as largely ineffective ‘paper parks.’ [8], [9] Over the last decade, evidence (reviewed below) has suggested, to the contrary, that protected areas are effective – only to be challenged, more recently, on methodological grounds. Meanwhile, from a social viewpoint, strict protected areas (which allow only conservation-related use) are sometimes viewed as effective in protecting biodiversity at the expense of excluding local inhabitants from access to forest resources [10]. Multiple use protected areas, which allow some sustainable use by local inhabitants, might potentially achieve both social and conservation goals – or fail at both [11],[12] – but quantitative studies on livelihood-conservation interactions are sparse [13].

Over the past decade, the growing availability of remotely sensed data on forest cover has facilitated quantitative studies of the impact of protected areas on tropical deforestation. Review papers include [12], [14], [15]; country-specific examples include Belize [16], Brazil [17], [18], [19], [20], Costa Rica [21], [22], [23], Honduras [24], Indonesia [25], [26], [27], [28], Madagascar [29], Peru [30] and Thailand [31]; global studies include [8], [32], [33]. These studies overwhelmingly report that protected areas are associated with lower deforestation rates.

The challenge for impact analysis is to construct a counterfactual: how much deforestation would have taken place if the forest in question had not been put under protection [34]; (see [15] for a detailed discussion and critical review of many studies). This requires controlling for social, economic and environmental factors that affect deforestation rate, and affect where protected areas are located. It is well-established that deforestation rates are lower in lands that are unattractive to agriculture: those that are remote from markets, have poor soils, high slopes, or heavy rainfall. (See [5] for a review.) But it is precisely these kinds of lands which governments might find it easiest to gazette for protection, where population density is low and powerful rural interests less likely to object [35]. And in fact, protected areas are disproportionately sited on lands characterized by higher slopes, higher elevations, and greater remoteness [36]. A naïve comparison of deforestation rates between these “low-pressure” areas and unprotected lands in general would give an inflated estimate of the effectiveness of the protected areas. On the other hand, [20] argue that some Brazilian forests have been designated as indigenous areas to protect them against very high deforestation pressures. Here, a comparison between protected and unprotected forests would yield an underestimate of the impact of protection.

Studies have approach this problem with different degrees of rigor. Some (e.g. [25], [30]) lack explicit controls, or assess deforestation in a protected area for which there is no comparable unprotected area [24]. Several use multivariate methods that explain the presence of deforestation, at the pixel level, as a function not just of protected area status, but also of slope, remoteness, and other determinants of both deforestation and of protected area placement [16],[26], [29]. A more sophisticated variant takes protected area status as endogenous, jointly modeling protection and deforestation via a bivariate probit model [37]. However, this approach requires nominating a variable which affects protection but not deforestation – a requirement difficult to fulfill.

More recently, some studies have used matching methods that are thought to be less sensitive to specification error than the multivariate econometric models. Matching methods seek to pair protected forest plots with unprotected but otherwise similar “control” plots. “Similarity” is defined on the basis of the control variables (such as slope and remoteness). As in the case of econometrics, the credibility of the models relies on the assumption that all significant confounding variables have been included.

For instance, [23] used matching methods to assess the deforestation-reducing impact of Costa Rica's system of protected areas. They found that protected areas on average did modestly reduce deforestation, but by substantially less than a naïve comparison of mean deforestation rates in protected versus unprotected areas [38]. qualifies this result, showing that Costa Rican parks had a greater protective effect in areas facing greater pressure, such as those close to the capital [28]. used propensity-score matching to assess the impact of protected areas on deforestation in Sumatra over 1990–2000, using high-resolution Landsat imagery. Compared to equivalent forests within protected areas, the study found deforestation rates to be 7.4 percentage points higher in buffer areas, and 24 percentage points higher in the landscape beyond.

At the global level, comprehensive evaluation has been hampered by inadequate data. There is no globally consistent, high-spatial resolution time series data for the entire tropical forest biome. Thus [8] relied on questionnaires aimed at protected area managers and [32] employed very coarse resolution (8 km square) remote sensing imagery to detect change; both covered only a subset of tropical protected areas. The study most comparable to ours is [33], which uses matching methods to assess protected area impacts across all biogeographical domains, not just the tropical forests. It uses two strategies to confront the lack of global deforestation data, each with some shortcomings. First, it uses natural land cover at a single point in time as a proxy for deforestation (i.e., change in land cover). Since it is possible that habitat clearance may have preceded (and motivated) the establishment of the protected area, a sensitivity test restricts the sample to pre-1980 protected areas. Second, it estimates land cover change over the period 2000–2005 by comparing two land cover datasets constructed with different methodologies. Since there is significant uncertainty in land cover classification at the 1 km resolution level, the difference between these two datasets may have a high noise/signal ratio, as the study acknowledges. It finds, overall, that the introduction of controls greatly diminishes but does not entirely nullify the estimate of protected area effectiveness. The global average difference in natural land cover in 2000 was about 2.5 percentage points; the difference in measured change between 2000 and 2005 was about 0.5 percentage points. The study finds protective impacts to be greater in flatter, less remote areas, and in the strictest forms of protected area (IUCN categories I and II).

The current study differs from [33] in several ways. It focuses on the tropical forest biome, a more homogeneous domain (from a biological and socioeconomic viewpoint) than the global set of protected areas. It uses what we will argue is a better proxy for land use change – namely, the occurrence of forest fires. And it partitions the set of protected areas along more policy-relevant lines, distinguishing strictly protected, multiple use, and indigenous areas. The study disaggregates results by continent and by remoteness from cities (a proxy for deforestation pressure). It finds in general that strict protected areas are effective, but less than a naïve assessment would indicate. In contrast, multiple use protected areas are in general more effective in reducing deforestation than strict protected areas, and are more effective than a naïve assessment would suggest.

## Methods

### Study area

The study is limited to developing countries (recipient countries of World Bank loans) and the extent of the tropical forest biome. These countries account for the bulk of deforestation and are potentially eligible for REDD participation. The biome—derived from the World Wildlife Fund's Terrestrial Ecoregions of the World [39]—contains the maximum spatial extent of the world's tropical and subtropical moist broadleaf forests.

**Figure 1:**
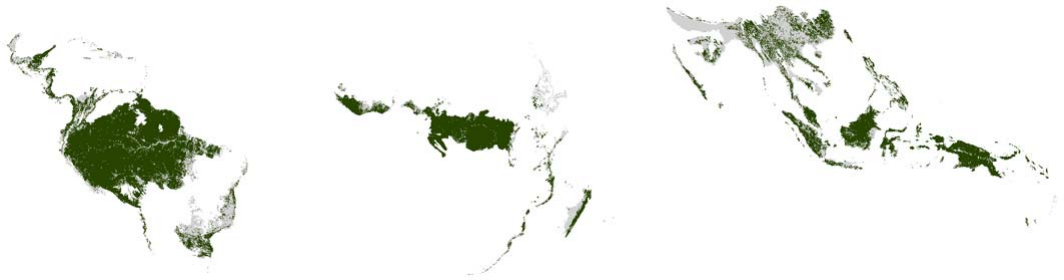
The tropical forest biome with standing forests in 2000. Gray: Tropical forest biome. Green: Standing forest, 2000.

Figure 1 shows the spatial intersection of these countries and the biome. The area in green is the maximum extent of the study area, covering 19.73 million km^2^. The biome is split across three continents; each will be analyzed separately. Papua New Guinea and Micronesia are considered part of Asia for this analysis.

Within this area, the extent of the remaining tropical forest in 2000 was extracted from two land cover data sources: Global Land Cover for the year 2000 [40] derived from ∼1 km resolution SPOT data and Percentage Forest Canopy Cover for 2000 [41] derived from ∼500 m MODIS data.

All 11 land cover classes from GLC2000 that contain forest or forest mosaics were extracted, along with all ∼1-km pixels where the average percent forest cover was greater than 25 percent [42]. This is a higher threshold than the 10 percent used in the FAO Forest Resource Assessment [43] and in a recent assessment of global forest protection [44]. One justification for using the 10 percent threshold in those global analyses was to capture woodland areas in Africa; however, these are not part of the tropical forest biome. Twenty-five percent was chosen to minimize the risk of including tropical woodlands/savannas and other land that was already largely cleared of forest, that was predominantly used for agriculture, and that could exhibit high fire activity that was not necessarily related to deforestation events.

Although both sources are well-documented research products, there are disagreements between the two datasets. To provide a conservative estimate of forest fire incidence, we use the intersection of the two forest covers within the boundaries of the biome covering 13.15 million km^2^ of tropical forest area in 2000. For reference, a tropical forest extent based on the MODIS data alone or on GLC2000 alone would amount to 15.13 million km^2^ or 14.51 million km^2^, respectively. Agreement between the two across the biome is 83.1 percent.

### Estimated deforestation fire activity: Outcome variable

The outcome variable was a binary measure: the presence or absence of at least one fire event on a given forested pixel (see Supplementary Information S1). Like other studies [19], we argue that forest fires are a reasonable proxy for tropical deforestation. In the tropics, non-anthropogenic fire is rare [45]. In Indonesia [46], found that fires were associated with land clearance in 8 of 9 study sites. In Amazônia, fires are associated with initial land clearance and with subsequent land management on the cleared plots [47]. (This motivates our choice of outcome measures not as the count of fires on a forest pixel, but the binary indication of whether one or more fires took place on the pixel during the observation period.) A comparison by Morton *et al.* [48] of screened ‘high-confidence’ fire detection with a deforestation measure based on high resolution Landsat imagery found that 87% of crop-related deforestation and 73% of pasture-related deforestation is associated with at least one such fire. Thus there is a small to moderate possibility of false negatives when using fires as a proxy for overt deforestation. The chance of false positives is minimized here by using Morton *et al.*'s high-confidence filter, which considers only fires occurring at night and daytime fires with >330 K brightness temperature in the 4 µm channel. While our indicator is imperfect, deforestation detection via visual interpretation of low-resolution imagery is also fallible, and even high-resolution Landsat imagery presents problems of interpretation, and of censoring due to cloud cover. The fire data and most imagery-based methods will fail to detect ‘cryptic’ forest degradation such as low-intensity logging.

Fire activity was estimated from spatially referenced remote sensing data on forest fires from the MODIS Active Fires dataset [49]. MODIS Active Fire data are provided on two satellite platforms, Terra from October 2000 and Aqua from July 2002, both to present day. Thus, there is partial coverage from October 2000 (two passes per day) and complete coverage from July 2002 (four passes per day), including both day and night passes.

High-confidence fires were extracted from more than 1 million MODIS fires scenes between 2000 and 2008 (http://modis-fire.umd.edu/Active_Fire_Products.html). Some 1.21 million 1-km pixels recorded at least one fire between October 2000 and January 2009 in the tropical forest biome and 0.70 million of these occurred in forested areas (Table 1). Of the 13.15 million 1-km tropical forest pixels, 5.31 percent had at least one fire event in that time frame.

**Table 1:** 1-km forest and fire pixel statistics in the tropical forest biome (2000–08).

Region	Forest pixels	Fire pixels	Fire rate
**Biome**	13,154,816	698,514	0.0531
**LAC**	6,989,019	365,074	0.0522
**Africa**	2,529,918	142,913	0.0565
**Asia**	3,635,879	190,527	0.0524

*Note:* LAC = Latin America and the Caribbean.

The outcome variable is a binary measure of forest fire activity per square km: was there at least one fire event in that pixel during 2000–08? This time period is reflected in the choice of covariables and the definition of the control/treatment groups below. The lack of coverage until October 2000 and then partial coverage until July 2002 implies that the binary measure here is slightly conservative as an estimate of fire-affected area.

Another dataset was considered as a proxy for tropical deforestation events: the recently released MODIS Collection 5 Burned Area Product, which includes global, monthly 500-meter (m) resolution maps of burn dates. A direct comparison between the active fire and burned area data for July 2001–June 2002 found that “many forest fires are detected by the active fire product but not by the burned area product” [50]. This higher detection rate, albeit including both medium- and high-confidence fires, and the fact that the burned area data are still provisional led to a preference for the active fire data over the burned area data as a proxy for tropical deforestation events.

The presence of one or more fires in a 1-km pixel cannot be directly translated into an estimate of deforested area. A fire event may represent anything from a small clearing of a single hectare to complete deforestation of the 1-km pixel. However, it can be assessed whether this fire presence/absence data can be used as a plausible proxy for deforestation activity in the tropical forest biome. We compared the binary measure of forest fire activity to deforestation as measured on a set of 183 Landsat scenes, used by Hansen *et al.* [42] for global imputation of deforestation. (Hansen et al use this high resolution data to calibrate global imputations based on lower resolution MODIS data. However, they caution against using the imputed data at the pixel level.) We plotted the area of fire activity for 2000–05 as a proportion of forest area against percent forest cover loss for 2000–05 per 18.5-km pixel (Supplementary Information S2). The analysis was repeated for 5 percent (top of figure) and 1 percent (bottom of figure) bins of forest cover loss.

There is a strong trend of increasing fire activity with increased loss of forest cover across the biome from 0 to 30 percent forest cover loss. The trend continues for higher forest cover loss percentages, but there are very few 18.5-km pixels (<0.2% of the tropical forest biome area) in these areas. Latin America and the Caribbean and Asia show the same clear trend as the whole biome, but the case is less clear for Africa. It should be noted that the remote sensing estimate of African deforestation differed drastically from the Forest Resources Assessment 2005 by the FAO [42], [43], so the deviation between the fire measures and the remote sensing measures may not be solely due to misclassification of the fire data.

From this it is reasonably sure that the chosen subset of active fires is a plausible proxy for deforestation events, especially in Latin America and Asia. The case is less convincing for Africa but is still plausible.

### Protected areas and IUCN management classes

The World Database on Protected Areas (WDPA) [51] is the source for protected area information. The WDPA is compiled by the United Nations Environment Programme and the IUCN, drawing on member organizations in 140 countries. While its accuracy depends on the reporting process [52], it is recognized as the most comprehensive and authoritative database available on protected areas and is commonly used in global studies of conservation (e.g. [33], [53]) It applies a rigorous, consistent, and detailed set of criteria to the identification and classification of protected areas [54]. Protected areas are defined as: “a clearly defined geographical space, recognized, dedicated and managed, through legal or other effective means, to achieve the long-term conservation of nature with associated ecosystem services and cultural values.” Protected area information, including boundaries (and PA center coordinates and area for PAs with unknown boundaries), designation date, IUCN protected area management classification, and status were extracted from the WDPA database for all protected areas that were inside or that intersected the tropical forest biome.

This list of protected areas includes all nationally (IUCN protected area management classes I through VI as well as unknown) and internationally (UNESCO MAB reserves, Ramsar sites, and World Heritage sites) recognized PAs and amounts to 4.13 million km^2^ of protected area within the biome, of which 3.62 million km^2^ is forested. (See Supplementary Information S3 for the IUCN definitions of management classes.)

Two treatment groups were considered, based on protected areas with boundary information. The first group consists of all protected areas that were designated pre-2000. The second group is restricted to protected areas that were designated between 1990 and 2000. Use of the restricted group allows us to examine the impact of more recently created protected areas and provides a check against the possibility of endogeneity in the matching variables.

Based on the IUCN categorization, PAs are classified as follows:

- Strict protection—IUCN classes I though IV
- Nonstrict or multi-use protection—IUCN classes V and VI
- Unknown protection—Nationally recognized but with no IUCN class
- Indigenous—A subset of the unknown class, but under indigenous stewardship.

**Figure 2:**
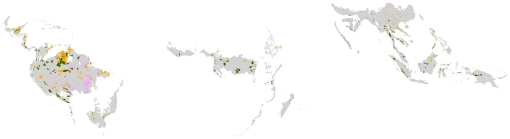
IUCN-designated protected areas established by 2000. Protected area category. Strict (IUCN I–IV) [green]. Multiuse (IUCN V, VI) [yellow]. Indigenous [pink].

*Strict protection* means areas that are designed specifically for nature protection. *Nonstrict protection* means that the areas have a multiple use management strategy. Category VI, for instance, comprises areas whose primary objective is “to protect natural ecosystems and use natural resources sustainably, when conservation and sustainable use can be mutually beneficial.” The *indigenous* group of protected areas occurs in Latin America, predominantly in Brazil, with a few areas in Panama and Colombia. Figure 2 shows the IUCN classified protected areas that were designated before 2000; the dominance of the protected tropical forest area in Latin America and the Caribbean is clear. There were 2,974 IUCN classified (IUCN classes I through VI, plus unknown) protected areas designated before 2000 in the tropical forest biome that contained at least 1 km^2^ of tropical forest.

The control groups are based on areas that have never been protected, up through 2008. We recognize that some forest areas that do not meet IUCN protected area criteria may benefit from other forms of legal protection – for instance, industrial forest concessions – so our comparisons may understate the effect of protection, broadly construed. Where boundary data was missing (for 22% of protected areas accounting for just 6% of total protected area extent), protected areas were represented by circles, with area equal to that of the protected area, centered around the point reported as the protected area location.

Summary statistics for tropical forest area and protected tropical forest area as of 2000 are shown below in Table 2. The number of observed tropical forest fire pixels and the tropical forest area for each region and protection group (pre-2000 areas only) are shown in Table 3.

**Table 2:** Total tropical forest protected (km^2^ and %) by protection class and region.

Area	Biome	Latin America and the Caribbean	Africa	Asia
**Forest Area**	13,154,816	6,989,019	2,529,918	3,635,879
**Protected Area**	3,619,941 (27.5)	2,719,301 (38.9)	411,761 (16.3)	488,879 (13.4)
Ia	166,892 (1.3)	152,650 (2.2)	1,425 (0.1)	12,817 (0.4)
Ib	21,207 (0.2)	10,415 (0.1)	1,097 (0.0)	9,695 (0.3)
II	740,910 (5.6)	482,193 (6.9)	127,902 (5.1)	130,815 (3.6)
III	57,837 (0.4)	47,140 (0.7)	483 (0.0)	10,214 (0.3)
IV	142,896 (1.1)	21,211 (0.3)	20,447 (0.8)	101,238 (2.8)
**Strict (I–IV)**	1,129,742 (8.6)	713,609 (10.2)	151,354 (6.0)	264,779 (7.3)
V	239,072 (1.8)	190,400 (2.7)	52 (0.0)	48,620 (1.3)
VI	799,854 (6.1)	716,626 (10.3)	26,069 (1.0)	57,159 (1.6)
**Multi-use (V–VI)**	1,038,926 (7.9)	907,026 (13.0)	26,121 (1.0)	105,779 (2.9)
**Unknown**	544,336 (4.1)	215,721 (3.1)	216,377 (8.6)	112,238 (3.1)
**Indigenous**	850,394 (6.5)	850,394 (12.2)	0 (0.0)	0 (0.0)
Other	56,543 (0.4)	32,551 (0.5)	17,909 (0.7)	6,083 (0.2)

*Note:* Numbers in parentheses are percentages.

**Table 3:** Forest and fire area (km^2^) and fire rates per region/protection group.

	Protection class	Forest pixels	Fire pixels	Fire rate*	fire rate relative to never protected**
**LAC**	Never	4,269,718	317,608	0.0744	
	Strict (I–IV)	472,676	7,597	0.0161	−0.0583
	Multi-use (V–VI)	533,549	16,245	0.0304	−0.0439
	Unknown	30,405	646	0.0212	−0.0531
	Indigenous	359,914	5,414	0.0150	−0.0593
**Africa**	Never	2,118,157	128,499	0.0607	
	Strict (I–IV)	142,169	2,538	0.0179	−0.0428
	Multi-use (V–VI)	21,705	654	0.0301	−0.0305
	Unknown	54,088	3,393	0.0627	0.0021
**Asia**	Never	3,147,000	172,212	0.0547	
	Strict (I–IV)	216,859	9,801	0.0452	−0.0095
	Multi-use (V–VI)	76,683	2,810	0.0366	−0.0181
	Unknown	35,315	495	0.0140	−0.0407

*The simple proportion of forest pixels that experience one or more fires.

**fire rate for unprotected pixels less fire rate for the PA category.

### Control variables

Variables describing terrain, climate, and remoteness were used to compare points in protected areas with ‘similar’ nonprotected points.

*Accessibility* to markets is a strong determinant of deforestation pressure [16]. A measure showing travel time to major cities in 2000 [55], [56] was used. This, the first such global measure, accounts for differential travel speeds on roads of different quality, railways, navigable rivers; and for off-road terrain, land cover, slope and elevation. Major cities are defined as having a population of 50,000 or more in 2000.

*Distance to road network* is a complementary measure of access to forest resources. A distance measure was created based on a vector road network extracted from the fifth edition of the Vector Smart Map Level 0 (VMap0) dataset. The primary source for the database is the 1∶1 million scale Operational Navigation Chart series. The reference period is 1979–99 [57] (The start date is debatable; the third edition of VMAP0, published 1997, also has a 20-year reference period—1974–94! The fifth edition was published in 2000, but given the minor changes after the first edition in 1992, it is unlikely to have much post-1990 data.)

*Distance to major cities* is a third proximity measure. A straight-line measure was created based on a point dataset of city centroids [58], using the same set of in the accessibility layer.

*Terrain* is a factor for land use suitability. Mild slopes and lower elevations are likely to be more accessible, more productive, more valuable, and thus more attractive for conversion to agriculture. As well as having a direct relation to suitability, slope and elevation are proxies for physical soil properties, and elevation is a proxy for temperature.

*Elevation* and *slope* were derived from the CSI-CGIAR version [59] of the 90-m resolution SRTM digital elevation model from NASA [60]. The CSI-CGIAR version of the data has filled in the data void areas with auxiliary digital elevation model data and topographically correct interpolation algorithms. The mean and variance of both slope and elevation were extracted at 1-km resolution.

*Rainfall* is another factor for land use suitability. Areas of extremely high rainfall are unlikely to be converted to agriculture, and the associated cloud cover and humidity preclude the use of fire activity as a reliable measure of deforestation.

Rainfall estimates were extracted from data provided by the Tropical Rainfall Monitoring Mission, specifically from the 3B42-TRMM-Adjusted Merged-Infrared Precipitation product [61]. This dataset provides monthly estimates of rainfall rates at a ¼-degree resolution. These rates were converted to millimeters (mm) per month, then aggregated into annual rainfall estimates and finally into an estimate of the average annual rainfall in mm for 2000–08.

Detailed *country* boundaries were extracted from the Global Administrative Areas database [62]. This information is used for exact matching to ensure that each control/treatment pair belongs to the same country.

*Ecoregions*, as defined by WWF, divide the world into 825 ecologically homogeneous areas [63]. Matching within ecoregions provides a more restrictive set of ‘equivalent’ forests than that enforced by matching on country and rainfall alone. For instance, Brazil is partitioned into 33 and Indonesia into 30 ecoregions. Since matching within ecoregions reduces the number of potential matches, we performed the analysis with and without this condition.

Summary statistics for all the above variables in the tropical forest and protected tropical forest areas are shown in Table 4. In general, protected tropical forest areas are more remote, have lower fire incidence rates, and have higher elevation/slope than the tropical forests as a whole.

**Table 4:** Summary statistics for variables in tropical forest areas.

		Forest Area			Protected forest area		
Region	Variable	Mean	St. Dev.	Median	Mean	St. Dev.	Median
**Biome**	**Travel time (minutes)**	1,353	1,401	817	1,678	1,528	1,181
	**Rainfall (mm)**	2,135	712	2,051	2,102	621	2,026
	**Dist. to cities (km)**	185	142	149	207	139	180
	**Dist. to roads (km)**	47	73	14	72	94	28
	**Fire pixels (proportion)**	0.053	0.224	0	0.026	0.158	0
	**Elevation (meters)**	410	483	245	449	510	281
	**Slope (degree)**	6.4	6.9	3	6.9	7.2	4
**LAC**	**Travel time (minutes)**	1,772	1,564	1,323	1,913	1,596	1,481
	**Rainfall (mm)**	2,197	571	2,186	2,099	499	2,060
	**Dist to cities (km)**	226	150	200	235	141	208
	**Dist to roads (km)**	76	87	44	94	101	54
	**Fire pixels (proportion)**	0.052	0.223	0	0.022	0.145	0
	**Elevation (meters)**	314	439	181	361	449	229
	**Slope (degree)**	4.8	5.8	2	5.5	6.3	3
**Africa**	**Travel time (minutes)**	646	563	486	889	652	736
	**Rainfall (mm)**	1,569	408	1,533	1,632	482	1,587
	**Dist to cities (km)**	145	92	131	166	97	160
	**Dist to roads (km)**	9	11	5	13	12	9
	**Fire pixels (proportion)**	0.057	0.231	0	0.030	0.170	0
	**Elevation (meters)**	493	362	441	581	533	446
	**Slope (degree)**	4.2	3.9	3	5.2	4.6	4
**Asia**	**Travel time (minutes)**	1,039	1,180	558	1,201	1,354	685
	**Rainfall (mm)**	2,410	885	2,365	2,436	905	2,438
	**Dist to cities (km)**	132	129	85	117	107	87
	**Dist to roads (km)**	18	28	7	19	29	9
	**Fire pixels (proportion)**	0.052	0.223	0	0.040	0.195	0
	**Elevation (meters)**	540	584	348	741	605	629
	**Slope (degree)**	11.3	8.1	11	14.0	7.9	14

### Data and sampling

All spatial data were projected to equal area sinusoidal projection, with a WGS84 datum and spheroid. Unless otherwise stated, raster resolution is 1 km. The relevant data from each data layer were extracted at 1-km spacing and stored in a PostgreSQL database (version 8.3), amounting to some 19 million records, one record per 1-km pixel. The matching analysis was split into three geographic regions: Latin America and the Caribbean, Africa, and Asia. A list of points that would be used to form the control and treatment groups was extracted from the database for each region. The list of points for the treatment group was based on a 10 percent random sample of points. The treatment points had to meet the following criteria:

- Were designated as protected pre-2000 based on protected area boundary information from the WDPA
- Classified as forest cover in 2000, based on the 11 land cover classes in GLC2000 that are forest or forest mosaic
- Met the 25 percent forest cover threshold from MODIS forest cover for 2000
- Fell into the relevant protection group (strict, multi-use, unknown, indigenous) for the cohort.

The two forest criteria reflect the conservative estimate of tropical forest area in 2000.

The corresponding control group was based on another random sample that was five times as large. The control points had to meet the following criteria:

- Had never been protected up to the end of 2008
- Classified as forest cover in 2000, based on the 11 land cover classes in GLC2000 that are forest or forest mosaic
- Met the 25 percent forest cover threshold from the MODIS forest cover for 2000.

The *never protected* area takes into account any form of recognized protection from the WDPA through the end of 2008 and including protected areas with information on their designation date. Those protected areas with boundary information are simply masked out. As noted, protected areas with a reported point location but no boundary information are treated as circles of the given area centered on their latitude/longitude coordinate, and those areas are also masked out.

### Analytic methodology

The analysis is on 1-km resolution data. The outcome variable is a binary measure of fire presence/absence from 2000–08 as a proxy for deforestation events. The treatment variable is protected/nonprotected. Randomly selected protected points (treatment group) are matched with similar control points, and the difference in deforestation (forest fire) rates is statistically evaluated. (For reviews and applications of matching methods, see [64], [65], [66]).

We use a combination of exact matching and nearest neighbor matching. Treatment and control points are matched exactly on country and on accessibility (travel time to nearest city, segmented by 15 minute increments). An additional five variables described earlier – average elevation, average slope, average rainfall (2000–08), distance to roads, and distance to cities – were used to select comparison points via nearest neighbor matching. We also conducted all analyses with and without the use of ecoregion as an exact matching variable. We use the commonly-employed Mahalanobis distance metric, a scale invariant measure of the multidimensional distance between two points. The algorithm randomly orders the treatment cases and for each one in turn selects the control case with the smallest distance. We matched with and without a 0.5 SD caliper. Use of a caliper (i.e. maximum acceptable distance) increases the quality of the matches but results in some unmatched points. Matching was performed with replacement and bias adjustment. The matching package [66] (version 4.7–6) running in the open source statistical program R (version 2.8.1) on MS Windows XP SP3 was used.

## Results

### Aggregate results

Table 5 shows the results of the matching analyses for all pre-2000 protected areas, alongside the crude (unmatched) estimates from Table 3. (In all cases the crude –comparing all protected pixels against all never protected pixels – and prematch rates – comparing an unmatched 10 percent sample of protected pixels against a similar proportion of never protected pixels – were very similar or identical, implying that the random sample was representative of the population.) Table 6 repeats, but uses the 1990–2000 protected areas as the treatment group. These tables and the subsequent discussion reflect results without the use of ecoregions as a matching variable. The use of ecoregions reduced the number of matching pairs but had little impact on the estimates. The ecoregion results are appended in Supplementary Information S4.

**Table 5:** Estimated impact on fire incidence (cumulative over 2000–08) comparing all pre-2000 protected areas against never protected areas.

			Without calipers		With calipers	
	Protection	Crude	Estimate [SE]	Pairs	Estimate [SE]	Pairs
**LAC**	**Strict**	−0.058	−0.027 [0.002]	46,015	−0.043 [0.001]	28,039
	**Multiuse**	−0.044	−0.048 [0.003]	52,505	−0.064 [0.002]	29,993
	**Unknown**	−0.053	−0.038 [0.010]	2,232	−0.023 [0.004]	511
	**Indigenous**	−0.059	−0.165 [0.003]	36,166	−0.163 [0.003]	28,482
**Africa**	**Strict**	−0.043	−0.010 [0.002]	13,507	−0.013 [0.001]	7,582
	**Multiuse**	−0.031	−0.030 [0.008]	1,592	§ −0.001 [0.004]	715
	**Unknown**	0.002	§ −0.010 [0.007]	4,980	§ 0.000 [0.004]	2,306
**Asia**	**Strict**	−0.010	−0.017 [0.003]	20,683	−0.020 [0.002]	12,101
	**Multiuse**	−0.018	−0.049 [0.006]	7,408	−0.043 [0.004]	4,319
	**Unknown**	−0.041	§ −0.010 [0.005]	3,528	−0.044 [0.003]	1,072

§ All estimates significant at p<0.001 except those marked with §.

Matching criteria exclude ecoregion.

**Table 6:** Estimated impact on fire incidence (cumulative over 2000–08) comparing 1990–2000 protected areas against never protected areas.

			Without calipers		With calipers	
	Protection	Crude	Estimate [SE]	Pairs	Estimate [SE]	Pairs
**LAC**	**Strict**	−0.065	−0.038 [0.003]	14,409	−0.077 [0.002]	5,749
	**Multiuse**	−0.030	−0.062 [0.004]	21,972	−0.075 [0.003]	15,032
	**Unknown**	−0.063	−0.026 [0.006]	889	too few points	80
	**Indigenous**	−0.061	−0.128 [0.004]	21,813	−0.127 [0.003]	15,276
**Africa**	**Strict**	−0.047	−0.022 [0.004]	2,730	−0.045 [0.004]	1,056
	**Multiuse**	−0.060	too few points	153	too few points	12
	**Unknown**	−0.059	−0.066 [0.008]	203	too few points	18
**Asia**	**Strict**	−0.022	−0.029 [0.005]	7,355	−0.031 [0.002]	2,536
	**Multiuse**	0.031	−0.067 [0.020]	1,832	−0.051 [0.008]	559
	**Unknown**	−0.049	−0.023 [0.006]	2,349	−0.070 [0.004]	569

Matching criteria exclude ecoregion.

*Note:* The full set of *balance* metrics and other outputs from these matching analyses are available on request.

Looking at Table 5, in the Latin America and the Caribbean region, the matched results for strict protection suggest a much lower level of avoided fire activity than the crude estimates. Nonetheless, protected areas reduced the incidence of forest fires by 2.7–4.3 percentage points against a mean loss of 5.8 percent (Table 3) over 2000–08. Multi-use protected areas appear to be more effective than strictly protected areas by approximately 2 percentage points, and this also translates into a larger area. “Unknown” is less effective, but the area involved is quite small. Indigenous areas are shown to reduce forest fire incidence by 16.3–16.5 percentage points, over two and a half times as much as the crude estimates (5.9 percent) and twice as effective as any other group in the matched results, with a greater estimated avoided fire-affected area than strict, multi-use, and unknown combined. Strictly protected areas in Africa are only one-quarter as effective (about a 1 percentage point impact) as the uncorrected estimates would suggest. The estimated impacts for multi-use areas are not robust: a significant 3 percent for the estimate without calipers, but 0 percent (with wide error bands) for the estimate with calipers. In Asia, strictly protected areas perform better than in the crude estimates, but multi-use protection is twice as effective as strict protection.

Table 6 estimates suggest that, with the exception of indigenous areas, protected areas designated between 1990 and 2000 offer better protection than pre-2000 protected areas as a whole, with improvements ranging from 1 to 3.5 percentage points, disregarding results with few matched pairs. In Latin America and the Caribbean, multi-use protected areas appear to be as effective or more effective than strictly protected areas, but indigenous areas are almost twice as effective as any other form of protection. In Asia, strictly protected areas perform better than in the crude estimates, but multi-use is twice as effective. In Africa, these recently established protected areas appear much more effective than the larger set considered in Table 5, with a robustly estimated impact of about 4.5 percentage points. There are too few points to estimate an impact of multi-use areas.

Table 7 summarizes the results. The range of estimates represents a robustness test—use of two kinds of matching procedures, and a more or less broad scope of protected areas, each with advantages and disadvantages. The conclusion that protected areas are effective at reducing fire incidence on forest is seen to be robust.

**Table 7:** Summary of estimated protected area reductions of fire incidence (percentage points).

Area	Mean fire incidence	Mean reduction due to strict protected areas	Mean reduction due to multi-use protected areas	Mean reduction due to indigenous areas
Latin America and Caribbean	7.4	2.7–4.3*3.8–7.7*	4.8–6.4*6.2–7.5*	16.3–16.5*12.7–12.8*
Africa	6.1	1.0–1.3*2.2–4.5*	(0.1)–3.0*Not calculated*	Not applicable
Asia	5.5	1.7–2.0*2.9–3.1*	4.3–4.9*6.7–5.1*	Not applicable

*Note:* Italics indicate estimates for protected areas established between 1990 and 2000. Parentheses indicate estimated increases in forest fire incidence.

Note that indigenous areas in Latin America are estimated to reduce fire incidence by more than 16 percentage points – yet the mean fire rate in never protected areas is just 7.4%. This suggests that indigenous areas tend to be located in areas of much-higher-than-average deforestation pressure. And indeed [36] show that multi-use protected areas are less prone to be located in low-pressure areas than are strictly protected areas.

To assess the importance of location when estimating the effectiveness of protection, the fire rate in the matched treatment and control groups is disaggregated by travel time. This is done only for the pre-2000 treatment group, as the 1990–2000 group often has too few points to allow disaggregation.

**Figure 3:**
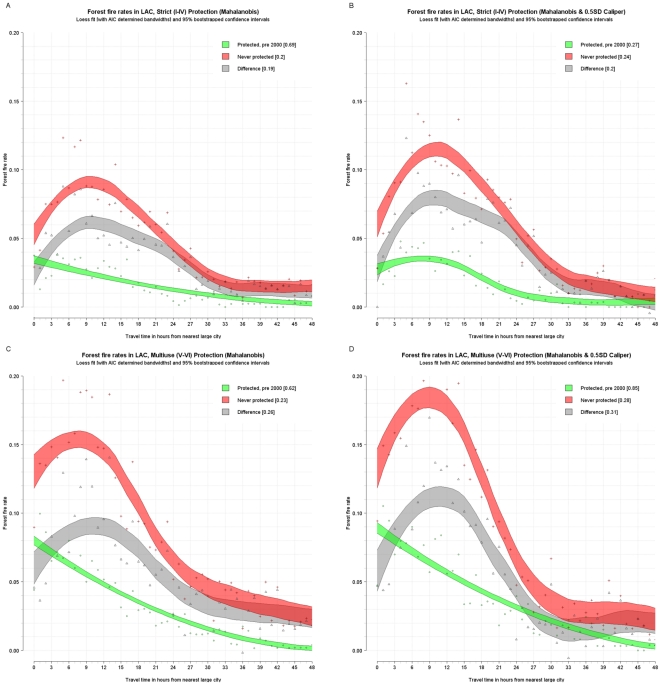
Unbiased estimated fire rates for tropical forests in Latin America and the Caribbean (with matching). Top – Strict protection in Latin America and the Caribbean, with Mahalanobis matching (A) and Mahalanobis matching with calipers (B). Bottom – Multi-use protection in Africa, with Mahalanobis matching (C) and Mahalanobis matching with calipers (D).

**Figure 4:**
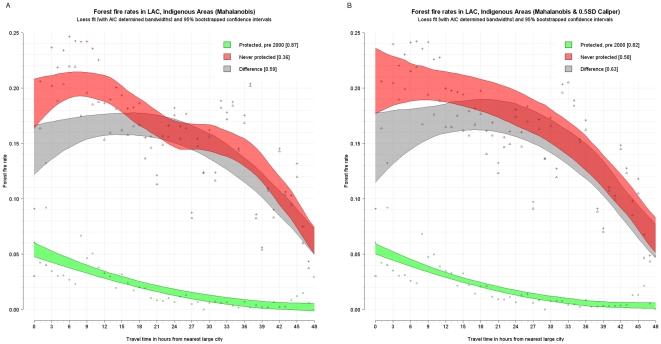
Unbiased estimated fire rates (red - never protected, green - protected, and grey – difference) for indigenous protection in Latin America and the Caribbean, with Mahalanobis matching (A) and Mahalanobis matching with calipers (B). *Note the change in scale on y axes*.

**Figure 5:**
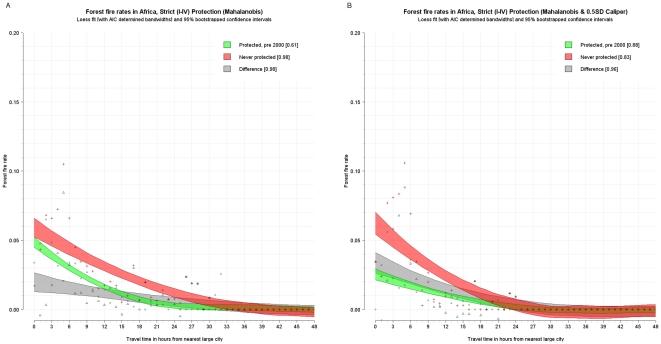
Unbiased estimated fire rates (red - never protected, green - protected, and grey – difference) for tropical forests in Africa with Mahalanobis matching (A) and Mahalanobis matching with calipers (B).

**Figure 6:**
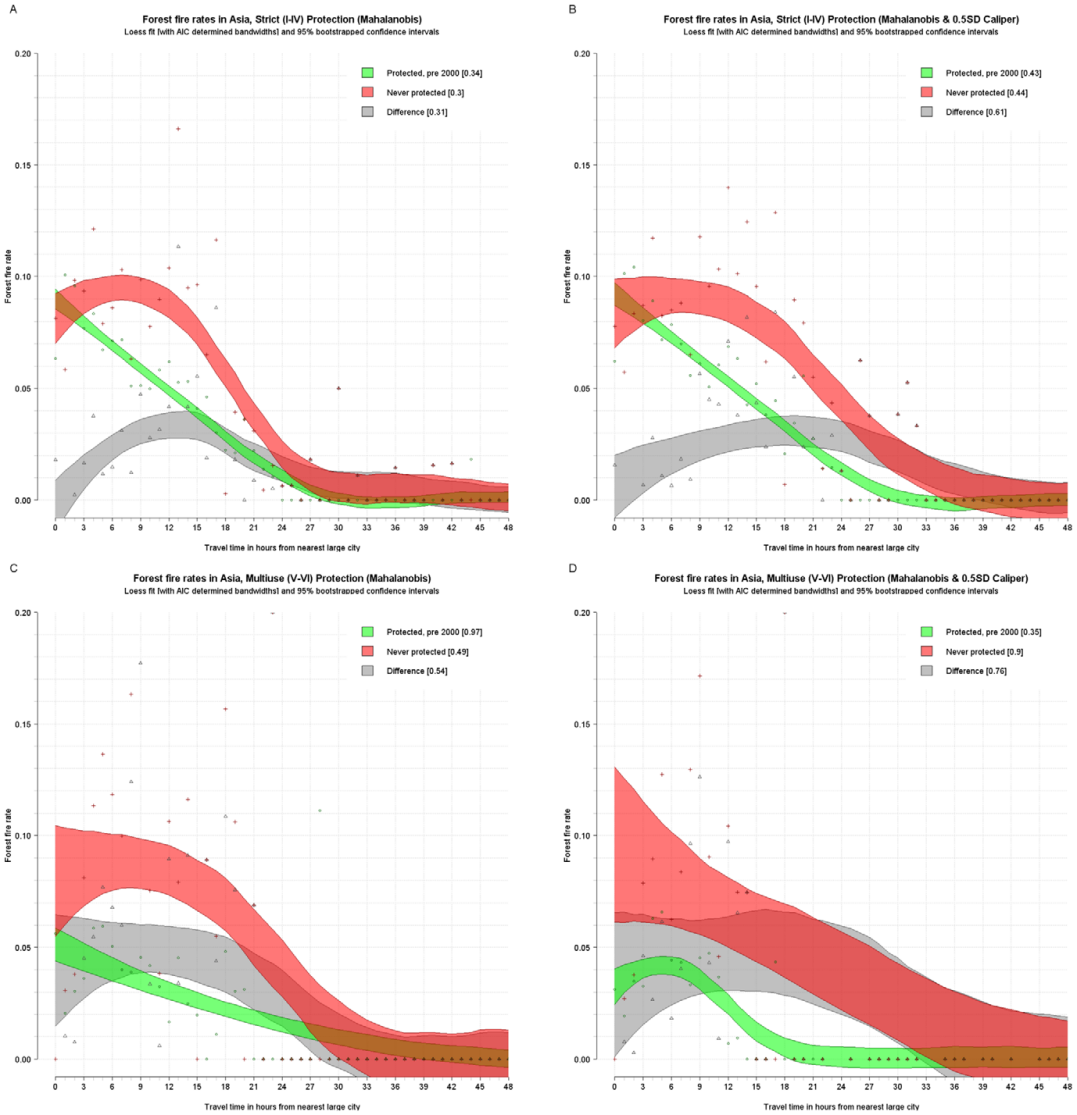
Unbiased estimated fire rates (red - never protected, green - protected, and grey – difference) for tropical forests in Asia (with matching). Top – Strict protection, with Mahalanobis matching (A) and Mahalanobis matching with calipers (B). Bottom – Multi-use protection, with Mahalanobis matching (C) and Mahalanobis matching with calipers (D).

The fire rate per travel time band was plotted and a loess curve was fitted through them using cross validation and Akaike's information criterion to determine the best fitting smoothing factor or bandwidth. Furthermore, the loess estimator (1,000 repetitions) was bootstrapped to determine 95 percent confidence intervals around the curve. (Given the small sample, these confidence intervals may be underestimated [67].) This was done for the fire rates from the matched control (never protected, red), and treatment data (protected pre-2000, green) and for the difference between the two (gray). This difference is essentially a disaggregated version of the estimates in Table 7 and provides an unbiased estimate of the avoided deforestation fires due to protection for different degrees of remoteness. The following figures show these confidence intervals around the loess curve as shaded polygons, as well as the points that they are fitted though. The results are reported for strict, multi-use, and indigenous areas for Latin America and the Caribbean (Figures 3 and 4), strict for Africa (there are insufficient pairs for multiuse to permit disaggregation) (Figure 5), and strict and multiuse for Asia (Figure 6), although the number of pairs for multiuse in Asia is just acceptable.

Some strong regularities emerge. First, in almost all cases, fire activity inside protected areas declines with increasing remoteness. Although the same is generally true for areas outside protected areas, in some cases (strict and multiuse in Latin America and the Caribbean and strict in Asia) the outside rate and hence effectiveness of protection increases with remoteness reaching a maximum at around 9–12 hours. Second, except for strict protection in Africa, protected areas generally have significantly lower fire rates than comparable nonprotected areas. However, this differential declines as remoteness increases. In other words, natural protection is as effective as formal protection in remote areas—at least for the moment. Third, in both Latin America and the Caribbean and Asia, nonremote multi-use areas are located in areas of higher deforestation pressure than strict areas. For instance, at one hour from cities in Latin America and the Caribbean, the controls for multi-use areas experience fire rates of about 16 percent whereas the controls for strict areas had fire rates of about 6 percent. Fourth, in Latin America and the Caribbean, fire rates are generally higher in multi-use than in strict protected areas, controlling for remoteness. Yet the impact of multi-use areas is greater than that of strict areas. At 1–12 hours from cities, for instance, multi-use protected areas reduce fire rates by about 6–12 percentage points, and strict protected areas reduce rates by only about 5 or 8 percentage points. Indigenous areas also have a very high absolute impact.

In Asia, the pattern is different. Controlling for distance, fire rates are higher in strict than in multi-use protected areas. Strict protected areas appear to be ineffective at deterring fires in nonremote areas. Their effectiveness increases with remoteness, peaking at about 12 hours distance from the city and declining thereafter. In contrast, multi-use protected areas are most effective in regions proximate to population centers.

In Africa, strict protected areas appear to have a modest impact. Estimates of the impact of multi-use areas are limited by a small sample and are not robust. The African estimates may be affected by outdated measurements of road proximity and remoteness from cities, since road conditions in parts of the Congo Basin have deteriorated since the reference period of the road maps used.

## Discussion

This paper uses forest fires as a proxy for deforestation and associated carbon release. Using global data for the tropical forest biome, it is apparent that protected areas have a substantially and statistically significantly lower incidence of forest fires than nonprotected areas, even after controlling for terrain, climate, and remoteness. The protective effect is greatest in nonremote areas (for Latin America and Africa) and areas of intermediate remoteness (Asia). Very remote areas have low fire rates even if unprotected—at least for the moment.

Importantly, it is clear that mixed-use protected areas—where some degree of productive use is allowed—are generally as effective or more effective than strict protected areas, especially in less remote areas with greater pressure for agricultural conversion and timber extraction. In Latin America, where indigenous areas can be identified, they are found to have extremely large impacts on reducing deforestation—much larger than a naïve, uncontrolled comparison would suggest. These results suggest that mixed-use and indigenous areas are disproportionately located in areas of higher deforestation pressure. This is noteworthy, given increasing attention to indigenous land rights.

From a policy viewpoint, these findings suggest that some kinds of land use restrictions—variations of protection—can be effective contributors to biodiversity conservation and climate change mitigation goals. The results suggest that indigenous areas and multi-use protected areas can help accomplish these goals, also suggesting some compatibility between environmental goals (carbon storage and biodiversity conservation) and support for local livelihoods. Zoning for sustainable use may be more politically feasible and socially acceptable than designation of strict protection in areas of higher population density and less remoteness. The results also reinforce findings from a field-survey based study of 84 protected areas in Asia and Africa, which found a positive association among biodiversity richness, forest support for livelihoods, and local people's participation in forest governance [13].

This analysis does not however attempt to measure “leakage” —the degree to which protection of one forest plot merely displaces conversion to another, unprotected plot. This is a more significant issue for carbon emissions than for biodiversity conservation, because the latter might be preferentially concerned with certain unique biodiversity locations whereas the former cares only about the density of carbon [68]. reviews theoretical and empirical studies of leakage and concludes that on both grounds leakage is far less than the 100 percent feared by critics. It points out that complementary policies (such as sponsoring crop intensification) could neutralize any leakage thought to arise from forest protection.

In addition, this analysis is unable to detect some kinds of forest degradation. Surreptitious removal of timber can result in biodiversity damage and lower carbon densities, but may not be detected through fire data.

Extension of this line of evaluation will be facilitated as better data become available. Improvements in remote sensing techniques and interpretation offer the prospect of more direct and precise measurement of deforestation and of forest carbon emissions. There is also a need to assemble, harmonize, and make public assessments of protected area management resources and practices in order to better understand the specific interventions that can contribute to reduced carbon emissions. Finally, there is a great need to complement land cover and land management measures with monitoring of human welfare and conditions in protected and unprotected forest areas.

It is important to stress that protected areas may be effective along other dimensions, even where there is little impact on current deforestation rates. This is especially true for protected areas established in remote regions with little current pressure for agricultural conversion. Such areas may already be effective in mitigating other threats, such as poaching of mammals and selective logging. Equally important, it is easier to reach consensus on the necessity and approach to protecting a forest before there are large economic pressures for conversion, often by people from outside the forest itself. A well-established protection regime may be better able to withstand pressures for unsustainable exploitation when the frontier arrives, as it eventually will in many currently remote places.

## Supporting Information

Supplementary Information S1 Fire activity 2000–2008 based on ‘high-confidence’ MODIS fires.
(TIF)

Supplementary Information S2 Fire area as a proportion of tropical forest area vs. tropical forest cover loss (2000–2005) A: 5% bins B: 1% bins.
(TIF)

Supplementary Information S3 IUCN protected area categories.
(DOCX)

Supplementary Information S4 Estimates using ecoregion as a matching variable.
(DOCX)
