# Supplementary figures and images for: Effectiveness of Strict vs. Multiple Use Protected Areas in Reducing Tropical Forest Fires: A Global Analysis Using Matching Methods
Source: PLoS One. 2011 Aug 16;6(8):e22722. doi: 10.1371/journal.pone.0022722 (PMC3156699; doi:10.1371/journal.pone.0022722)

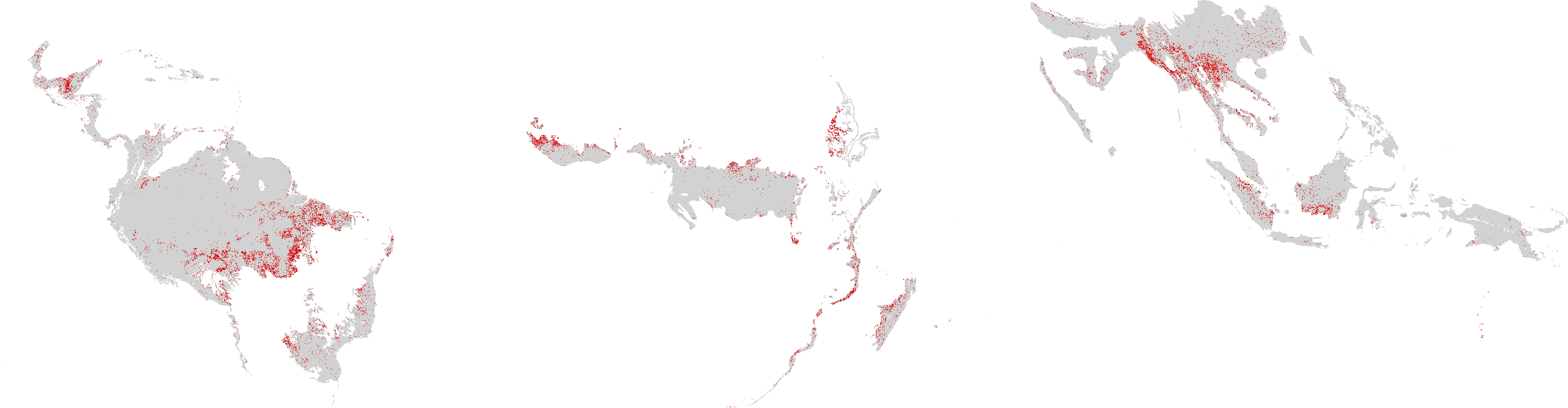

Supplement: Supplementary Information S1 — Fire activity 2000–2008 based on ‘high-confidence’ MODIS fires. (TIF) [file pone.0022722.s001.tif]

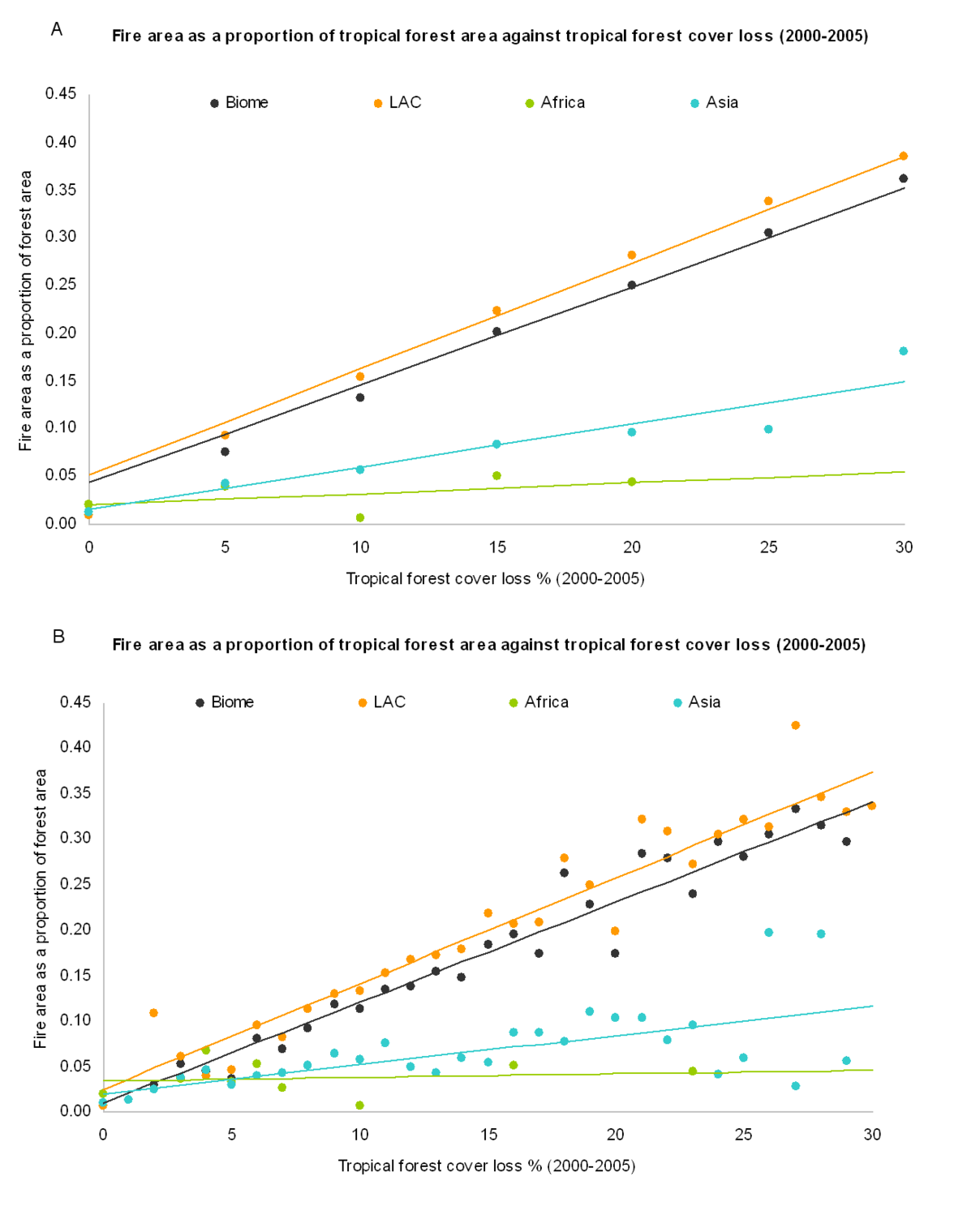

Supplement: Supplementary Information S2 — Fire area as a proportion of tropical forest area vs. tropical forest cover loss (2000–2005) A: 5% bins B: 1% bins. (TIF) [file pone.0022722.s002.tif]
