# Supplementary material for: Effectiveness of Strict vs. Multiple Use Protected Areas in Reducing Tropical Forest Fires: A Global Analysis Using Matching Methods
Source: PLoS One. 2011 Aug 16;6(8):e22722. doi: 10.1371/journal.pone.0022722 (PMC3156699; doi:10.1371/journal.pone.0022722)
Supplement: Supplementary Information S3 — IUCN protected area categories. (DOCX) [file pone.0022722.s003.docx]

**Supplementary information S3**

**IUCN Categories of Protected Areas**

**CATEGORY Ia: Strict Nature Reserve:** Protected area managed mainly for science.
**Definition:** Area of land and/or sea possessing some outstanding or representative ecosystems, geological or physiological features and/or species, available primarily for scientific research and/or environmental monitoring.

**CATEGORY Ib: Wilderness Area:** Protected area managed mainly for wilderness protection.
**Definition:** Large area of unmodified or slightly modified land and/or sea retaining its natural character and influence, without permanent or significant habitation, which is protected and managed so as to preserve its natural condition.

**CATEGORY II:** **National Park:** Protected area managed mainly for ecosystem protection and recreation.

**Definition:** Natural area of land and/or sea designated to (a) protect the ecological integrity of one or more ecosystems for present and future generations; (b) exclude exploitation or occupation inimical to the purposes of designation of the area; and (c) provide a foundation for spiritual, scientific, educational, recreational, and visitor opportunities, all of which must be environmentally and culturally compatible.

**CATEGORY III:** **Natural Monument:** Protected area managed mainly for conservation of specific natural features.

**Definition:** Area containing one or more specific natural or natural/cultural feature that is of outstanding or unique value because of its inherent rarity, representative or aesthetic qualities, or cultural significance.

**CATEGORY IV:** **Habitat/Species Management Area:** Protected area managed mainly for conservation through management intervention.

**Definition:** Area of land and/or sea subject to active intervention for management purposes to ensure the maintenance of habitats and/or to meet the requirements of specific species.

**CATEGORY V:** **Protected Landscape/Seascape:** Protected area managed mainly for landscape/seascape conservation and recreation.

**Definition:** Area of land, possibly with coast and sea, where the interaction of people and nature over time has produced an area of distinct character with significant aesthetic, ecological, and/or cultural value, and often with high biological diversity. Safeguarding the integrity of this traditional interaction is vital to the protection, maintenance, and evolution of such an area.

**CATEGORY VI:** **Managed Resource Protected Area:** Protected area managed mainly for the sustainable use of natural ecosystems.

**Definition:** Area containing predominantly unmodified natural systems, managed to ensure long-term protection and maintenance of biological diversity, while providing a sustainable flow of natural products and services to meet community needs.
