# Supplementary material for: Effectiveness of Strict vs. Multiple Use Protected Areas in Reducing Tropical Forest Fires: A Global Analysis Using Matching Methods
Source: PLoS One. 2011 Aug 16;6(8):e22722. doi: 10.1371/journal.pone.0022722 (PMC3156699; doi:10.1371/journal.pone.0022722)
Supplement: Supplementary Information S4 — Estimates using ecoregion as a matching variable. (DOCX) [file pone.0022722.s004.docx]

**Supplementary information S4**

**Estimates with ecoregion as a matching variable.**

**Table S3-1 All pre-2000 protected areas**

|  |  |  | **Without calipers** | | **With calipers** | |
| --- | --- | --- | --- | --- | --- | --- |
|  | **Protection** | **Crude** | **Estimate [SE]** | **Pairs** | **Estimate [SE]** | **Pairs** |
| **LAC** | **Strict** | -0.058 | -0.027 [0.002] | 41,613 | -0.050 [0.001] | 21,951 |
|  | **Multiuse** | -0.044 | -0.041 [0.002] | 47,080 | -0.062 [0.002] | 23,413 |
|  | **Unknown** | -0.053 | § -0.011 [0.005] | 1,146 | § -0.004 [0.003] | 385 |
|  | **Indigenous** | -0.059 | -0.187 [0.004] | 34,054 | -0.184 [0.003] | 19,434 |
|  |  |  |  |  |  |  |
| **Africa** | **Strict** | -0.043 | § -0.006 [0.002] | 12,851 | -0.012 [0.001] | 7,280 |
|  | **Multiuse** | -0.031 | § -0.001 [0.006] | 1,353 | § 0.006 [0.003] | 606 |
|  | **Unknown** | 0.002 | § 0.003 [0.007] |  | § 0.004 [0.004] | 2,256 |
|  |  |  |  |  |  |  |
| **Asia** | **Strict** | -0.010 | -0.016 [0.003] | 18,223 | -0.015 [0.002] | 8,152 |
|  | **Multiuse** | -0.018 | -0.045 [0.005] | 6,493 | -0.044 [0.003] | 3,102 |
|  | **Unknown** | -0.041 | -0.015 [0.004] | 2,310 | -0.047 [0.002] | 533 |

§ All estimates significant at p<0.001 except those marked with §.

**Table S3-2: Protected areas established 1990-2000**

|  |  |  | **Without calipers** | | **With calipers** | |
| --- | --- | --- | --- | --- | --- | --- |
|  | **Protection** | **Crude** | **Estimate [SE]** | **Pairs** | **Estimate [SE]** | **Pairs** |
| **LAC** | **Strict** | -0.065 | -0.029 [0.003] | 11,221 | -0.057 [0.002] | 4,000 |
|  | **Multiuse** | -0.030 | -0.064 [0.004] | 20,133 | -0.078 [0.003] | 11,866 |
|  | **Unknown** | -0.063 | § 0.006 [0.003] | 439 | too few points | 63 |
|  | **Indigenous** | -0.061 | -0.141 [0.004] | 19,255 | -0.128 [0.003] | 9,747 |
|  |  |  |  |  |  |  |
| **Africa** | **Strict** | -0.047 | -0.021 [0.004] | 2,271 | -0.043 [0.004] | 1,023 |
|  | **Multiuse** | -0.060 | too few points | 83 | too few points | 9 |
|  | **Unknown** | -0.059 | too few points | 136 | too few points | 10 |
|  |  |  |  |  |  |  |
| **Asia** | **Strict** | -0.022 | -0.023 [0.005] | 5,845 | -0.034 [0.002] | 1,527 |
|  | **Multiuse** | 0.031 | -0.062 [0.014] | 1,136 | -0.077 [0.006] | 354 |
|  | **Unknown** | -0.049 | -0.034 [0.006] | 1,328 | -0.059 [0.003] | 250 |

§ All estimates significant at p<0.001 except those marked with §.
